# Prevalence of allergen sensitization in 1000 adults in Saskatchewan

**DOI:** 10.1186/s13223-017-0181-1

**Published:** 2017-02-08

**Authors:** Stacey D. Lok, Beth E. Davis, Donald W. Cockcroft

**Affiliations:** 1 Department of Internal Medicine, University of Saskatchewan, 103 Hospital Drive, Saskatoon, SK S7N 0W8 Canada; 2 Division of Respirology, Critical Care and Sleep Medicine, Department of Medicine, University of Saskatchewan, 5th Floor Ellis Hall, 103 Hospital Drive, Saskatoon, SK S7N 0W8 Canada

**Keywords:** Hypersensitivity, Asthma, Skin tests, Epidemiology

## Abstract

**Background:**

The prevalence of sensitization varies geographically based on multiple environmental factors including humidity. The aim of this study was to determine the prevalence of atopy in symptomatic adults. More importantly we aimed to obtain a regional statistic of sensitization to common allergens given Saskatchewan’s dry climate.

**Methods:**

One thousand consecutive symptomatic adults were screened for atopy via skin prick test over 10 years (2006–2016) in the Division of Respirology. An atopic screen was performed with twenty common aeroallergens by a single investigator, Dr. D. Cockcroft. A positive test was considered to be a wheal ≥3 mm and markedly positive reactions ≥8 mm were also documented.

**Results:**

The prevalence of atopy by means of a positive skin test (≥3 mm) was 45.5%. The prevalence of one or more markedly positive reactions (≥8 mm) was 29.5% of the total population. The most frequent sensitization was to cat dander (58.2%), followed by mixed grass (32.1%), and birch (26.8%). Dust mite sensitization was 22.4% and mouse 6.2%. A positive epidemiology screen for cat/grass/mite would have incorporated 82.0% (n = 373) of subjects with positive skin tests. Those who failed the cat/grass/mite screen were mainly sensitized to trees (n = 34), molds (n = 22), weeds (n = 7), and animals (n = 8).

**Conclusions:**

There is a high prevalence of cat sensitization in Saskatchewan, much higher than recorded in other centers internationally. This is likely due to a high proportion of cat ownership. The prevalence of mite sensitization is lower than those mentioned at other centres likely due to Saskatchewan’s dry climate. The significance of the rate of markedly positive reactions (≥8 mm wheal) when compared to humid areas with higher burden of mite is unknown. There is a low prevalence of roach also likely due to the dry climate and mouse sensitization was low but still identified as a significant indoor allergen. A cat/grass/mite screen may be useful with a 82.0% sensitivity.

## Background

Allergen sensitization varies according to multiple environmental factors including humidity [1]. The purpose of this study was to gain an appreciation of the prevalence of atopy in symptomatic patients in Saskatchewan and develop a regional statistic of common allergens in Saskatoon and the surrounding area. It was hypothesized that our allergen prevalence may differ from other regions based on our dry climate and sensitization to dust mite and roach may be lower for this reason. Based on previous studies performed in Saskatchewan the prevalence of atopy in the general population was observed in 38.3% (≥3 mm wheal) [2]. The prevalence of markedly positive reactions (≥8 mm wheal) is also of interest as it has not been previously studied in our region and may have a bearing on severity of disease. A proposed cat/grass/mite screen [3] was also assessed to determine the utility in using these three skin tests as a screen for detecting atopy in our population.

## Methods

One thousand consecutive skin prick test panels (Table 1) were collected from the years 2006–2016 on primarily adult patients. The concentration of the allergen, the allergen itself, and the supplier remained unchanged throughout this time period. All testing was performed by a single investigator, Dr. D. Cockcroft using the epicutaneous technique on the volar surface of the forearm with a Hollister Stier Lancet (#8372ZA, purchased from Jubilant Hollister Stier 3525 N Regal St, Spokane, WA 99207, USA) at the Royal University Hospital in patients with symptoms suggestive of atopy, rhinitis, or asthma (Table 2). Data were collected including age, gender, and symptoms of cough, dyspnea, rhinitis. Any patients with an underlying diagnosis of CF/bronchiectasis and asthma were recorded. A protocol of twenty common aeroallergens (Table 1) were used as well as a negative control (sodium chloride 0.9%/glycerine 50%) and a positive control (histamine 1 mg/mL base, 2.75 mg/mL phosphate, glycerine 50%). A positive skin prick test was considered to be ≥3 mm wheal larger than any reaction to the negative control and markedly positive reactions as ≥8 mm. One or more positives using a hypothetical 3 skin test epidemiology screen (cat/grass/mite) was also documented.

**Table 1:** Skin test allergen concentrations and contents

Aspergillus	1:10 equal parts niger/terreus/repens/oryzae
Cladosporium	1:10
Dreshslera	1:10
Alternaria	1:10
Birch	1:20
Manitoba Maple	1:20
Mixed grass	100,000 BAU/mL
Mixed weeds	1:20
Poplar	1:20
Ragweed	1:20
Willow	1:20
Wheat dust	1:10
Cat	10,000 BAU/mL cat pelt extract
Cattle	1:20
Dog	1:20
Horse	1:20
Mouse	1:20
House dust mite	5000 AU/mL, equal parts Der p/Der f
Cockroach	1:20 two equal parts American/German roach
Feathers	1:20 equal parts chicken/duck/goose
Control	sodium chloride 0.9% glycerine 50%
Histamine	1 mg/mL base, 2.75 mg/mL phosphate, glycerine 50%

Allergens were purchased from ALK Abello. ALK Pharmaceuticals Inc, Mississuga, Ontario

*Der p* Dermatophagoides pteronyssinus, *Der f* Dermatophagoides farina

**Table 2:** Population demographics and prevalence of atopy

	Total	Atopic	Non-atopic
n	1000	455	545
Age (years)			
Mean (SD)	46.5 (18)	41.5 (17.8)	50.6 (17.2)
Female n (%)	567 (56.7)	252 (55.4)	315 (57.8)
Diagnoses n (%)			
Asthma	658 (66)	355 (78)	303 (56)
Cough	138 (14)	33 (7)	105 (19)
Dyspnea	121 (12)	33 (7)	88 (16)
Rhinitis	44 (4)	16 (4)	28 (5)
CF/bronchiectasis	39 (4)	18 (4)	21 (4)

Age: paired t test p < 0.0001, female: Chi squared p = 0.44, diagnoses: Chi squared p < 0.0001

## Results

After analyzing skin tests in 1000 individuals, 455 patients (45.5%) were determined to be atopic (one or more skin wheal ≥3 mm). Of these 1000 patients, 295 (29.5%) had one or more markedly positive tests with wheals measuring ≥8 mm. The remainder of the 545 tests yielded 515 negative skin tests, 24 with no response to histamine and 5 with dermatographisms and no positives.

The mean age of the total population was 46.5 years, with a mean age of 41.5 years in the atopic, and 50.6 years in the non-atopic patients. Females accounted for 56.7% of population with 55.4% of the atopic and 57.8% of the non-atopic population being female. The primary diagnosis and reason for testing in the 1000 individuals with descending order of prevalence was asthma, isolated cough, dyspnea not yet diagnosed, rhinitis, and cystic fibrosis/bronchiectasis.

**Fig. 1:**
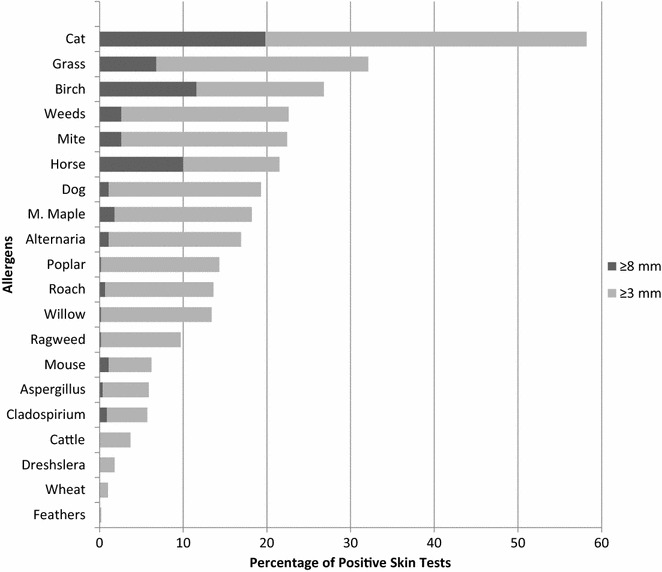
Profile of sensitization to allergens. A positive epidemiology screen for cat/grass/mite would have incorporated 82.0% (n = 373) of positive skin tests. Those with negative cat/grass/mite responses showed positive responses to trees (n = 34), molds (n = 22), weeds (n = 7), animals (n = 8), cockroach (n = 5), and feathers (n = 1)

The frequencies of positive (≥3 mm) and markedly positive (≥8 mm) skin tests in the 455 atopic patients are shown in Fig. 1. The most frequent sensitization was cat dander (58.2%), followed by mixed grass (32.1%), and birch (26.8%). Dust mite sensitization was found in 22.4% and mouse in 6.2%.

A positive epidemiology screen for cat/grass/mite would have incorporated 82.0% (n = 373) of positive skin tests. Those with negative cat/grass/mite responses showed positive responses to trees (n = 34), molds (n = 22), weeds (n = 7), animals (n = 8), cockroach (n = 5), and feathers (n = 1).

## Discussion

The primary objective of this study was an epidemiological surveillance of the prevalence of sensitization in symptomatic adults in the Saskatoon region. Given the large sample size and collection of data from ten consecutive years, an appropriate sample was felt to be achieved. Some limitations to the sample included the data of rural or urban location of the patient was not collected. Given environment is an important contributor to sensitization profiles [1] this information may have been of interest.

One of the most intriguing components to our results was the extremely high rates of cat sensitization in our sample. Sensitization to cat was found in 58.2% of individuals with positive skin prick tests. When comparing this to other studies the rates found were 17.7% in a journal from Chile [4] and 24.3% from a cumulative report from several major centres in the United States including Baltimore, New York, Watertown, Augusta, California, St Louis, and Washington [5]. A possible explanation for the higher rate of cat sensitization observed in our sample may be related to the higher rate of cat ownership and exposure compared to other urban centers with less animal ownership. Saskatoon has 4433 registered cats as of September 2016 through the City of Saskatoon [6]. However, it was not as prominent as other cities in the surrounding area including Regina who has 4757 [7] registered cats and 39,367 in Calgary [8]. According to a 2013 market survey, 57% of Canadians have a household pet and 35% of Canadian households own a cat [9]. To our knowledge, this prominent rate of cat allergy has not been documented in a Canadian centre prior to this study.

Our rate of dust mite sensitivity prevalence (22.4%) was found to be significantly lower than those mentioned at other centres. Knowing that approximately one-third (38.3%) of the Saskatchewan population is atopic [2], our mite sensitization prevalence can be extrapolated to be significantly lower in the general population. Studies published in Hungary (27.8%) [10] and 17% in suburban and 26% in urban Belgium [11] demonstrate similar rates sensitization in their general population to what we found in Saskatchewan’s atopic population. A study performed in the United Kingdom reports a sensitization rate of as high as 82% to Der f in their atopic population [12].

The hypothesis that dust mite sensitization in Saskatchewan is comparatively low due to our dry climate is supported by a succession of publications from Australia which studied two cities in New South Wales(NSW) [13–15]. All papers included one region with a humid climate and high rates of house dust mite burden and another region with a drier climate and lower burden of house dust mite. In the coastal, more humid climate of Lismore, NSW, sensitization rates in the general population were reported to be as high as 33.3% as compared to 28.3% in the drier inland city of Wagga Wagga, NSW [14].Wagga Wagga’s humidity was reported to be 29–67% in another paper documenting an 11.3% sensitization to Der p in the general population. This was significantly lower when compared to Newcastle, NSW, another coastal city with a humidity of 62–74% and a reported 20.3% Der p sensitivity.

Despite the difference in humidity and burden of mite in Lismore, NSW(humid) vs. Moree/Narrabri, NSW(dry), sensitization rates were similar 28.6 and 26.4% respectively, however the humid region was associated with more severe bronchial responsiveness despite similar rates of sensitization [13]. This raises the question of whether the rate of markedly positive dust mite skin prick test reactions are lower in dry climates like Saskatchewan (2.6%) when compared to more humid regions. Unfortunately, the rate of markedly positive reactions is not frequently documented nor is its relationship to humidity, especially for sensitization to dust mite and roach which we know thrive in humid conditions.

The role of mouse allergen has more recently been addressed as an important contributor to non-occupational allergic respiratory disease [16]. Findings in the inner-city asthma study (ICAS) measured sensitization of inner-city school age children with asthma and found 22% of children had a positive skin test to mouse [16]. Levels of mouse allergen in the home differ substantially with higher rates in inner city dwellings and lower rates in rural areas. [16] Our study found a lower sensitization to mouse which was not surprising as there is a considerable population of rural patients serviced in our health region, however our rates were still quite significant at 6.2%.

The utilization of a cat/grass/mite screening tray (3 individual skin tests) was also analyzed in this study and we found this to have a sensitivity of 82% for identifying atopy. This is a fairly reasonable sensitivity, however to be used as a screening test a higher sensitivity would be desired. The highest proportion of atopic subjects who failed the screen showed sensitivity to tree (44% of those who failed, n = 34). Given that birch was found to be the third highest sensitivity, perhaps if repeated a cat/grass/mite/birch epidemiological screening may have a high enough sensitivity to be used exclusively as a screening test in our health region.

## Conclusions

In summary, a prevalence of atopy was obtained in and around Saskatoon, Saskatchewan. The highest prevalence in this region is to cat dander (58.2% of atopics) which is higher than previously documented in other regions internationally. The rate of dust mite sensitivity (22.4%) was found to be lower than those mentioned in other centres and is thought to be secondary to Saskatchewan’s dry climate. The prevalence of markedly positive reactions is low (2.6%), anecdotally this is thought to be lower than more humid regions such as Eastern Canada. There was a low sensitization to mouse (6.2%), however this was non-negligible and certainly an important contributor to non-occupational allergic respiratory disease. A cat/grass/mite epidemiological screen in our health region was helpful but is not sensitive enough to be used exclusively as a screening test for atopy. This screen may have been more successful in a region with higher rates of mite sensitization.

## Abbreviations

CF: cystic fibrosis
M.Maple: Manitoba Maple
SD: standard deviation
BAU: bioequivalent allergy unit
AU: allergy units
Der p: Dermatophagoides pteronyssinus (house dust mite)
Der f: Dermatophagoides farina (house dust mite)
